# Incidence of cytomegalovirus infection among the general population and pregnant women in the United States

**DOI:** 10.1186/1471-2334-7-71

**Published:** 2007-07-02

**Authors:** Fernando AB Colugnati, Stephanie AS Staras, Sheila C Dollard, Michael J Cannon

**Affiliations:** 1 Disciplina de Nutrição e Metabolismo, Departamento de Pediatria, Universidade Federal de São Paulo, São Paulo, Brazil; 2 Centers for Disease Control and Prevention, Atlanta, Georgia, 30333, USA; 3 Rollins School of Public Health, Emory University, Atlanta, Georgia, 30322, USA

## Abstract

**Background:**

Cytomegalovirus (CMV) is a common opportunistic infection among HIV-infected individuals, a major source of serious complications among organ-transplant recipients, and a leading cause of hearing loss, vision loss, and mental retardation among congenitally infected children. Women infected for the first time during pregnancy are especially likely to transmit CMV to their fetuses. More children suffer serious disabilities caused by congenital CMV than by several better-known childhood maladies such as Down syndrome or fetal alcohol syndrome

**Methods:**

Using CMV seroprevalence data from the nationally representative Third National Health and Nutrition Examination Survey, we estimated CMV incidence among the general United States population and among pregnant women. We employed catalytic models that used age-specific CMV seroprevalences as cumulative markers of past infections in order to derive estimates of three basic parameters: the force of infection, the basic reproductive rate, and the average age of infection. Our main focus was the force of infection, an instantaneous per capita rate of acquisition of infection that approximates the incidence of infection in the seronegative population.

**Results:**

Among the United States population ages 12–49 the force of infection was 1.6 infections per 100 susceptible persons per year (95% confidence interval: 1.2, 2.4). The associated basic reproductive rate of 1.7 indicates that, on average, an infected person transmits CMV to nearly two susceptible people. The average age of CMV infection was 28.6 years. Force of infection was significantly higher among non-Hispanic Blacks (5.7) and Mexican Americans (5.1) than among non-Hispanic Whites (1.4). Force of infection was significantly higher in the low household income group (3.5) than in the middle (2.1) and upper (1.5) household income groups. Based on these CMV incidence estimates, approximately 27,000 new CMV infections occur among seronegative pregnant women in the United States each year.

**Conclusion:**

These thousands of CMV infections in pregnant women, along with the sharp racial/ethnic disparities in CMV incidence, are compelling reasons for accelerating research on vaccines and other interventions for preventing congenital CMV disease. Nevertheless, the relatively low force of infection provides encouraging evidence that modestly effective vaccines and rates of vaccination could significantly reduce CMV transmission.

## Background

Cytomegalovirus (CMV) is a common opportunistic infection among human immunodeficiency virus (HIV)-infected individuals, a major source of serious viral complications among organ-transplant recipients, and a leading cause of hearing loss, vision loss, and mental retardation among congenitally infected children. In fact, more children suffer serious disabilities caused by congenital CMV than by several better-known childhood maladies such as Down syndrome or fetal alcohol syndrome [1].

Like other herpesviruses, primary CMV infection is followed by the establishment of lifelong latent infection from which periodic reactivation is common [2,3]. Symptoms are usually absent during primary infection and reactivation, but CMV can be shed in various bodily secretions, particularly urine and saliva [4]. CMV is transmitted person-to-person via close non-sexual contact, sexual activity, breastfeeding, blood transfusions, and organ transplantation [4]. For pregnant women, important sources of infection include sexual activity and contact with the urine or saliva of young children, especially their own children [5-7].

Congenital CMV infection is most likely to occur following a primary infection in the mother during pregnancy [8]. However, maternal CMV reactivation or reinfection with a different CMV strain can also lead to fetal infection [8]. Approximately 10 percent of congenitally infected infants are symptomatic at birth, and of the 90 percent who are asymptomatic, 10–15 percent will develop symptoms over months or even years [9].

Incidence of primary CMV infections has been estimated only in small or specialized populations, such as pregnant women or day care providers. The most comprehensive study of CMV incidence was carried out by Griffiths and colleagues in the United Kingdom [10], in which they estimated that more than three seronegative women per 100 seroconvert each year. However, their study was limited to pregnant women and was hospital-based rather than population-based. Robust, nationally representative estimates of CMV incidence are essential for 1) assessing the burden of primary CMV infection in the United States population, especially among pregnant women; 2) examining whether there are racial/ethnic disparities in primary maternal infection rates, which might be responsible for racial/ethnic disparities in congenital infection rates; and 3) evaluating how effective a vaccine or other intervention must be in order to reduce the incidence of congenital CMV disease. To obtain estimates of CMV incidence in the United States, we employed mathematical models that used age-specific CMV seroprevalences from the Third National Health and Nutrition Examination Survey (NHANES III).

## Methods

### Study population and design

NHANES III was conducted from 1988 to 1994 and provides nationally representative estimates of the health and nutritional status of the civilian, noninstitutionalized population of the United States. In order to produce population-representative estimates, NHANES III used a multistage, stratified, clustered sample design and generated sample weights proportional to the probability of participant selection. All our analyses used the NHANES III sample weights and sample design variables to correct the CMV seroprevalence point estimates for population representativeness and the interval estimates for the multistage complex sample design. The study protocol was approved by the authors' institutional review board. More details about NHANES III can be found in the official documentation [11]. Serologic testing for CMV immunoglobulin G (IgG) was conducted as described previously [12].

The main focus of our models of CMV incidence was the age range 12–49 years. Over 90 percent of participants in this age range had sera available for CMV testing (N = 11,859) so that seroprevalence estimates were representative of the United States population. More importantly, this age range included women of childbearing age and so has key relevance for congenital CMV disease. Although surplus sera was only available for approximately 70 percent of 6–11 year-olds (N = 2,679), we also ran models in this age group to assess whether incidence rates differed by race/ethnicity. Nationally-representative CMV seroprevalence estimates were not available for children less than six years old.

### Description of models

Here we give an overview of the models of CMV incidence. A more detailed description is provided in the Appendix. We employed catalytic models [13,14] that used age-specific CMV seroprevalences as cumulative markers of past infections in order to derive estimates of three basic parameters: the force of infection, the basic reproductive rate, and the average age of infection. The force of infection is the instantaneous per capita rate of acquisition of infection [13] and will be expressed in this article as the number of primary CMV infections per 100 seronegative persons per year. The basic reproductive rate is a function of the force of infection and is the average number of secondary infections produced when one infected individual is introduced into a host population where everyone is susceptible. The average age of infection is also a function of the force of infection and is the age at which an individual in a given population typically acquires a specific infection. We considered parameter differences to be statistically significant when corresponding confidence intervals did not overlap.

**Figure 1:**
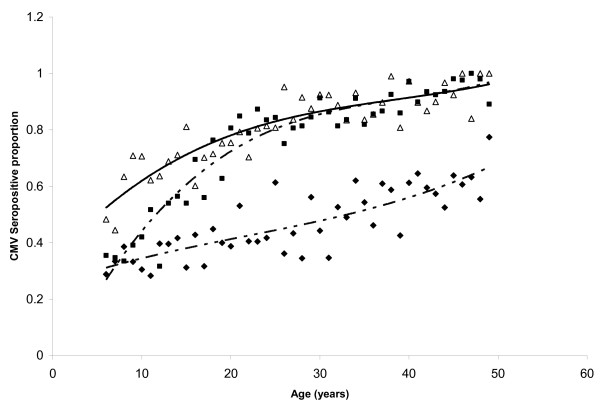
**Age-distribution of cytomegalovirus (CMV)-seropositive proportion among U.S. women**. Age-distribution of cytomegalovirus (CMV)-seropositive proportion among women in the National Health and Nutrition Examination Survey (NHANES) III, stratified by race/ethnicity. Observed seroprevalences: △-Mexican American, ◆-Non-Hispanic (NH) Whites, ■- NH Blacks. Adjusted third degree polynomials: ------- Mexican Americans, -------- NH Whites, --------- NH Blacks.

Force of infection can be estimated as time-dependent, age-dependent, or both. Since our data were taken from a single, cross-sectional survey, we could not model time dependence. To evaluate age-dependence, we visually inspected the slope of the age-specific seroprevalence graph (see Appendix). We observed no extreme departures from linearity for the overall population, with the slope appearing fairly constant as a function of age. However, because we saw age-dependent changes in slope within some subpopulations (e.g., Figure 1), we used piece-wise log-linear models that allowed the slope to vary between the age groups 6–11, 12–19, and 20–49 years. With the exception of this modification for the subgroup analysis, our final models were the time- and age-independent ones proposed by Griffiths et al. [10] for modeling CMV incidence, where the force of infection is estimated as the slope of the log-linear regression line having the seronegative prevalence as the response variable and age as the explanatory variable. For all models age was treated as a continuous variable.

The models made the following assumptions: CMV infection does not affect the mortality rate; seroprevalence in newborns equals zero; the death rate is type I, meaning everyone survives until a specific age, after which the survival probability is zero; and every person in the population is equally susceptible (*i.e.*, homogeneous mixing).

### Variables

We estimated the model parameters for the entire United States population and for specific population groups stratified by sex, race/ethnicity, and/or household income. Race/ethnicity was a self-reported variable that consisted of non-Hispanic Whites, non-Hispanic Blacks, Mexican Americans and Others. As recommended in NHANES III documentation [15], Others was excluded from the analyses because the sample size was too small and encompassed a diverse mix of race/ethnicity. Household income was divided into low, medium, and high, as defined previously [11,12].

### Estimating risk of CMV infection during pregnancy

We estimated risk of CMV infection for seronegative women during pregnancy as *risk *= 100 × [1 - *e*(^-*rate*×*time*^)], where rate was the force of infection per 100 women per year and time was the duration of pregnancy [16]. We multiplied this risk by the proportion of women who are CMV seronegative to obtain risk of CMV infection during pregnancy for the entire population (i.e., seronegative and seropositive) of women. We then multiplied the risk of infection in the entire population of women by the average number of live-birth pregnancies per year for the years 1988–1994 [17]. This product represented the estimated annual number of women with a primary CMV infection during pregnancy.

## Results

The overall force of CMV infection in 12–49 year-olds in the United States was 1.6 per 100 persons per year (Table 1). The associated basic reproductive rate of 1.7 indicates that, on average, an infected person transmits CMV to nearly two susceptible people. The average age of CMV infection was 28.6 years. Among 12–49 year-olds, CMV force of infection was significantly higher among non-Hispanic Blacks (5.7) and Mexican Americans (5.1) than among non-Hispanic Whites (1.4) (Table 1). These differences were reflected in the average age (in years) of infection, which was 16.3 for non-Hispanic Blacks, 17.5 for Mexican Americans, and 29.3 for non-Hispanic Whites. Force of infection was significantly higher in the low household income group (3.5) than in the middle (2.1) and upper (1.5) household income groups.

**Table 1:** CMV force of infection, basic reproductive rates, and average age of infection among persons 12–49 years old in the United States.

	Force of Infection (95% CI)*		Basic reproductive rate (95% CI)		Average age of infection in years (95% CI)	
Entire U.S. population	1.6	1.3–1.9	1.7	1.5–1.8	28.6	27.3–29.4
Sex						
Female	1.8	1.3–2.2	1.7	1.5–1.9	28.0	26.2–29.9
Male	1.5	1.1–1.8	1.6	1.4–1.8	29.1	27.7–30.6
Race/Ethnicity						
Non-Hispanic Black	5.7	5.1–6.2	4.1	3.7–4.4	16.3	15.1–17.5
Mexican American	5.1	4.3–5.6	3.7	3.2–4.2	17.5	15.8–19.4
Non-Hispanic White	1.4	1.1–1.8	1.6	1.4–1.7	29.3	27.9–30.6
Income per family size						
Low	3.5	2.8–4.5	2.7	2.1–3.2	21.9	19.0–24.9
Middle	2.1	1.6–2.6	1.9	1.7–2.2	26.7	24.9–28.6
High	1.5	1.1–1.9	1.6	1.4–1.8	28.9	27.5–30.4

*Number of infections per 100 susceptible persons per year. We considered parameter differences to be statistically significant when corresponding confidence intervals did not overlap. CI, confidence interval.

**Figure 2:**
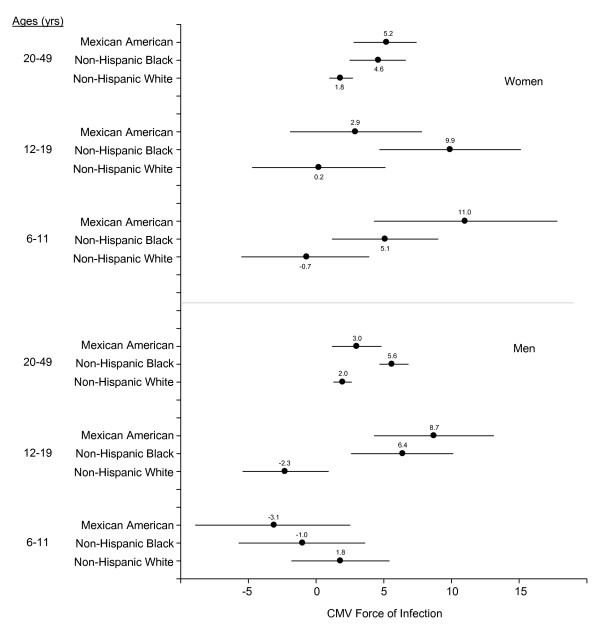
**Cytomegalovirus (CMV) force of infection**. Cytomegalovirus (CMV) force of infection stratified by sex, age group (6–11, 12–19, and 20–49 years), and race/ethnicity. Circles represent point estimates and lines represent 95% confidence intervals. Negative values for force of infection can occur because the models treat the CMV seroprevalences as if they come from a single cohort followed over time, when in fact they are age-specific seroprevalences of a population at a single point in time. Thus, in the younger ages where the sample sizes are smaller, it is possible for an older age group to have a somewhat lower seroprevalence than a younger age group, which can lead to a negative value for force of infection. We considered force of infection differences to be statistically significant when corresponding confidence intervals did not overlap.

We observed considerable variation in force of infection when we stratified by age and sex (Figures 1 and 2). Among adolescent girls (ages 12–19 years), non-Hispanic blacks had a substantially higher force of infection (9.9) than the other groups. In contrast, among pre-adolescent girls (ages 6–11 years), Mexican Americans had the highest force of infection (11.0). Among adolescent boys (ages 12–19 years), force of infection was highest in non-Hispanic blacks (6.4) and Mexican Americans (8.7).

Among seronegative women ages 20–49 years, risk of primary CMV infection during a full-term pregnancy was estimated to be 1.38 percent among non-Hispanic Whites, 3.40 percent among non-Hispanic Blacks, and 3.85 percent among Mexican Americans (Table 2). However, among 12–19 year-old seronegative women, risk was much higher for non-Hispanic blacks (7.33 percent) than for Mexican Americans (2.21 percent) and for non-Hispanic whites (0.15 percent). The estimated annual number of women ages 12–49 experiencing primary CMV infection during pregnancy was approximately 27,000. Most of these infections occur in non-Hispanic Whites because they are the largest racial/ethnic group in the U.S. However, non-Hispanic Blacks and Mexican Americans, especially those under age 30, are disproportionately likely to have pregnancies in which they experience primary CMV infections.

**Table 2:** Risk and frequency of CMV primary infection during pregnancy in the United States.

Ages (years)	% Seronegative	Risk among seronegative women/100 pregnancies*	Risk for all women/100 pregnancies	No. live-birth pregnancies (100's)†	No. women with primary infection during live-birth pregnancies
Non-Hispanic White					
12–19	61.0	0.15	0.09	2320	209
20–29	56.7	1.38	0.78	12140	9469
30–39	49.4	1.38	0.68	9120	6201
40–49	38.9	1.38	0.54	510	275
				
Subtotal				24090	16154
Non-Hispanic Black					
12–19	42.6	7.33	3.12	1330	4150
20–29	17.8	3.40	0.61	3060	1867
30–39	13.4	3.40	0.46	1350	621
40–49	5.3	3.40	0.18	80	14
				
Subtotal				5820	6652
Mexican American					
12–19	30.1	2.21	0.67	1220	817
20–29	17.5	3.85	0.67	3990	2673
30–39	10.5	3.85	0.40	1700	680
40–49	6.8	3.85	0.26	100	26
				
Subtotal				7010	4196
Total				36940	27002

*Risk is computed as 100* [1-exp(-rate × time)], where rate is force of infection per 100 women per year (0.2/100 and 1.8/100 for NH-White, 9.9/100 and 4.5/100 for NH-Black, and 2.9/100 and 5.1/100 for Mexican American) and time is duration of pregnancy in years, i.e., 40/52 = 0.77 years. †From National Vital Statistics Report, Vol. 49, No. 4, June 6, 2001 [17].

## Discussion

Robust estimates of the frequency of new CMV infections are essential for understanding and preventing viral transmission. This study provides the first estimates of CMV incidence that are based on population-representative data. We found that among CMV-seronegative individuals aged 12–49 in the United States, nearly one in 60 seroconverts each year.

This relatively low force of infection indicates that CMV is less easily transmitted than some other infections, such as measles or rubella. For these infections, high vaccine efficacy and coverage are required in order to interrupt transmission [18]. In contrast, a CMV vaccine would not need to have such high efficacy and coverage to substantially prevent CMV transmission. Griffiths et al. [10], who estimated forces of CMV infection of 3.1–3.5/100 persons/year in the United Kingdom, showed that modest rates of vaccination (~60 percent) would be able to eradicate CMV infection from the human population. Our estimates, which are similar but even lower overall (force of infection = 1.6/100 persons/year), provide further evidence that modestly effective vaccines and rates of vaccination could significantly reduce CMV transmission.

Our models identified large racial/ethnic disparities in the frequencies of new CMV infections. The force of infection for CMV was considerably higher in non-Hispanic Blacks and Mexican Americans than in non-Hispanic Whites. The nearly three-fold differences in risk of primary CMV infection among seronegative women could be responsible for much of the racial/ethnic disparities in rates of infants born with congenital CMV [19]. Racial/ethnic differences were especially pronounced among adolescent girls (ages 12–19 years), among whom primary infection was 50 times more likely in seronegative non-Hispanic blacks and 15 times more likely in seronegative Mexican Americans than in non-Hispanic whites. These higher forces of infection (i.e., incidence in seronegative individuals) suggest that CMV is circulating more frequently in these racial/ethnic groups. Thus, seropositive, pregnant non-Hispanic blacks and Mexican Americans may be at a higher risk of suffering re-infection with a different strain of CMV, which also places their infants at risk of symptomatic congenital CMV [8]. These disparities indicate that interventions, such as vaccines or education campaigns, may need to be tailored to meet the needs of different racial/ethnic groups and different age groups.

In addition to race/ethnicity, low household income was a risk factor for CMV infection. People with low household income may be more likely to have a larger family and experience crowding, thus facilitating CMV transmission via close contact. However, because force of infection was more strongly associated with race/ethnicity than with household income, high-risk racial/ethnic groups may have a higher prevalence of additional factors related to CMV transmission, such as increased exposure to CMV while caring for young children. A more detailed analysis of risk factors for CMV infection in NHANES III can be found in Staras et al. [12].

Among women ages 20–49 years, force of infection appeared to be independent of age, suggesting that risk of infection during pregnancy is fairly constant during these ages, and that interventions to prevent congenital CMV must target all women of childbearing age. CMV had a higher force of infection than infections transmitted primarily via sex or injection drug use, such as herpes simplex virus type 2 (HSV-2) or hepatitis B virus (HBV). This suggests either that CMV is more easily transmissible through such behaviors [20] or, more likely, that CMV is transmitted via other, additional routes. Given that CMV has been shown to be transmitted via urine or saliva during close, non-sexual contact, it is likely that this sort of transmission plays a major role in the dynamics of CMV infection [7].

We estimated that each year in the United States more than 27,000 pregnant women experience primary CMV infection and are thus at high risk of giving birth to a child with congenital CMV infection. This estimate does not include any fetal losses that may have been caused by primary CMV infection, nor does it include the many pregnancies affected by CMV reactivation or reinfection among seropositive women. The burden of primary CMV infections during pregnancy falls disproportionately on disadvantaged women--those of low income and racial/ethnic minorities. Furthermore, teenaged minority women are at especially high risk of primary CMV infections during pregnancy, due to their high prevalence of susceptibility, high force of infection, and high pregnancy rates.

The risk of primary CMV infection during pregnancy among seronegative women is similar to previous estimates [4]. For seronegative women, CMV infection represents one of the highest risks for fetal damage that they experience during pregnancy [21]. Because CMV transmission is potentially preventable [1], CMV antibody screening prior to or near the beginning of pregnancy should be evaluated as a means of identifying women at high risk for having congenitally infected infants. Studies should pursue whether knowledge of high risk status is a useful motivational tool for modifying behaviors, such as hand hygiene, for reducing risk of infection [22]. Such screening may also lead to the administration of CMV hyperimmuneglobulins or antiviral drugs for prevention or therapy of fetal infection and disease [23,24].

In this study the modeling assumptions appeared to have been reasonably satisfied. On a population level, CMV infection does not contribute significantly to mortality among infected individuals. Nearly all members (=99 percent) of the population are susceptible at birth, and infection is believed to induce life-long immunity. The type I death-rate cut-off was chosen as 70 years to approximate the U.S. life expectancy during the years that NHANES III was conducted, but modifying the cut-off had little effect on the model results. The assumption of homogeneous mixing is unlikely to be completely true, but because CMV infection is common and has multiple transmission modes, susceptible individuals are likely to have similar risks of exposure to CMV.

An important limitation of our models was that the data were from a single, cross-sectional study so that time trends were not able to be addressed. Thus, high CMV seroprevalence in cohorts of older people might not reflect current incidence and could cause the models to overestimate the force of infection [12]. We sought to minimize this potential bias by focusing most of our analyses on a limited age range (12–49 years). It is also important to note that our younger, age-specific force of infection estimates (i.e., for ages 6–11 and 12–19 years) were imprecise, with wide confidence intervals. Furthermore, the models implicitly assumed that seroprevalence was monotonically increasing with age, as if this cross-sectional study were a cohort study in which seroprevalence was measured at various ages of follow-up. However, this assumption was violated for some of the younger subpopulations. As a result, we occasionally obtained negative estimates for the force of infection (Figure 2), although these estimates were not statistically different from zero.

The calculations of risk of primary infection during pregnancy required several assumptions, one of which was that the force of infection was the same for pregnant and non-pregnant women. Women who are pregnant may have fewer sex partners (and thus lower risk of exposure to CMV) during pregnancy; on the other hand, pregnant women may be more likely than non-pregnant women to be exposed to young children (a group that frequently sheds CMV). Pregnant women may also have a higher risk of acquiring infections because of pregnancy-induced immune depression [25].

Based on our models, we would estimate that more than one million United States women have experienced primary CMV infections during pregnancy since CMV was first isolated 50 years ago [26,27]. A substantial proportion of these infections would have led to congenital infections, leaving thousands of children with lifelong disabilities. Children from disadvantaged racial/ethnic groups are likely to have been disproportionately impacted. These many affected children are a compelling argument for accelerating research on vaccines and other interventions for the prevention of congenital CMV [28].

## Conclusion

Each year, thousands of CMV infections occur in pregnant women in the United States, putting numerous unborn babies at risk for serious disabilities. Incidence of CMV infection in pregnant women is not distributed evenly, but exhibits sharp racial/ethnic disparities, especially affecting non-Hispanic blacks and Mexican Americans. Because of the magnitude of the problem and its associated health disparities, there is an urgent need to accelerate research on vaccines and other interventions for preventing congenital CMV disease. Nevertheless, the low incidence of CMV infection relative to other vaccine-preventable infections provides encouraging evidence that modestly effective vaccines and rates of vaccination could significantly reduce CMV transmission.

## Competing interests

The author(s) declare that they have no competing interests.

## Authors' contributions

FABC designed and carried out the mathematical modeling and statistical analyses and drafted the manuscript. SASS participated in the design and implementation of the CMV testing of the NHANES III specimens. SCD coordinated and supervised the CMV testing of the NHANES III specimens. MJC conceived of the study, participated in its design and coordination, and helped to draft the manuscript. All authors read and approved the final manuscript and revised it critically for important intellectual content.

## Appendix

To estimate CMV incidence by using the force of infection, we used the catalytic model approach described in Farrington [14] and Anderson [13]. We began by assuming that the force of infection was age-dependent, so that

**Figure 3:**
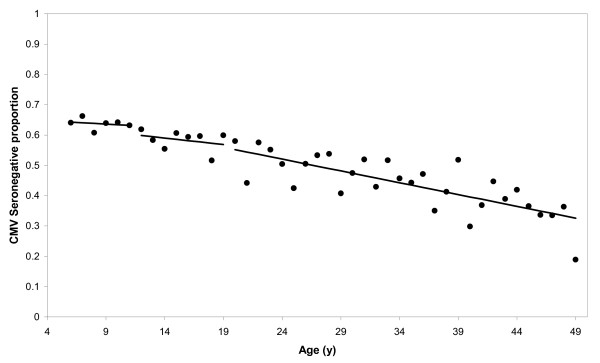
Example of piece-wise log-linear model among non-Hispanic black women.

S−(a)=e−∫0aλ(x)dx,
MathType@MTEF@5@5@+=feaafiart1ev1aaatCvAUfKttLearuWrP9MDH5MBPbIqV92AaeXatLxBI9gBaebbnrfifHhDYfgasaacH8akY=wiFfYdH8Gipec8Eeeu0xXdbba9frFj0=OqFfea0dXdd9vqai=hGuQ8kuc9pgc9s8qqaq=dirpe0xb9q8qiLsFr0=vr0=vr0dc8meaabaqaciaacaGaaeqabaqabeGadaaakeaacqWGtbWudaahaaWcbeqaaiabgkHiTaaakiabcIcaOiabdggaHjabcMcaPiabg2da9iabdwgaLnaaCaaaleqabaGaeyOeI0Yaa8qCaeaaiiGacqWF7oaBcqGGOaakcqWG4baEcqGGPaqkcqWGKbazcqWG4baEaWqaaiabicdaWaqaaiabdggaHbGdcqGHRiI8aaaakiabcYcaSaaa@42AD@

where *a *is age and *S*^-^*(a) *is the age distribution for the seronegatives. To assess the shape of the integral above we proceeded as Farrington, by visual inspections. *λ*(*x*)was evaluated as an exponential decay function and as a polynomial of third or lesser degree. Despite permitting *λ*(*x*) to be a complicated function, force of infection was approximately constant as a function of age (i.e., force of infection was age-independent). Therefore, we used the log-linear approach where the force of infection is the slope of the regression model (i.e., *λ*(*x*) equals the constant λ) given by *ln*(*S*^-^(*a*)) = -(*β*_0 _+ *λa*). This model, used by Griffiths [10] to estimate CMV force of infection, also seemed to fit the NHANES III data in most cases, where *β*_0 _plays the role of the natural logarithm of the age-adjusted seronegative proportion. We made one modification to this model when we estimated force of infection within subgroups: λ was treated as constant within pieces of the age range, namely, 6–11 years, 12–19 years, and 20–49 years (Figure 3).

With the age-independent assumption, the average age of infection, *A*, and the basic reproductive rate, *R*_0_, were estimated by:

A=1λ(1−(1+λL)exp⁡(−λL)1−exp⁡(−λL)),R0=λL1−exp⁡(−λL),
 MathType@MTEF@5@5@+=feaafiart1ev1aaatCvAUfKttLearuWrP9MDH5MBPbIqV92AaeXatLxBI9gBaebbnrfifHhDYfgasaacH8akY=wiFfYdH8Gipec8Eeeu0xXdbba9frFj0=OqFfea0dXdd9vqai=hGuQ8kuc9pgc9s8qqaq=dirpe0xb9q8qiLsFr0=vr0=vr0dc8meaabaqaciaacaGaaeqabaqabeGadaaakeaafaqabeqacaaabaacbmGae8xqaeKaeyypa0ZaaSaaaeaacqaIXaqmaeaaiiWacqGF7oaBaaWaaeWaaeaadaWcaaqaaiabigdaXiabgkHiTiabcIcaOiabigdaXiabgUcaRiab+T7aSjab=XeamjabcMcaPiGbcwgaLjabcIha4jabcchaWjabcIcaOiabgkHiTiab+T7aSjab=XeamjabcMcaPaqaaiabigdaXiabgkHiTiGbcwgaLjabcIha4jabcchaWjabcIcaOiabgkHiTiab+T7aSjab=XeamjabcMcaPaaaaiaawIcacaGLPaaacqGGSaalaeaacqWFsbGudaWgaaWcbaGaeGimaadabeaakiabg2da9maalaaabaGae43UdWMae8htaWeabaGaeGymaeJaeyOeI0IagiyzauMaeiiEaGNaeiiCaaNaeiikaGIaeyOeI0Iae43UdWMae8htaWKaeiykaKcaaiabcYcaSaaaaaa@63D4@

where *L = 70 *is the threshold age for the type I death rate.

When estimating force of infection for different subgroup categories, one category was chosen to be the referent category and the others were represented by indicator variables and were included in the models with interaction for age. For example, in the case of race/ethnicity, which had 3 categories and White as the referent category, the model was:

ln(*S*^-^(*a*)) = -(*β*_0 _+ *λ*_0_*a *+ *β*_1_*δ*[*White *- *Black*] + *β*_2_*δ*[*Mexican*] + *λ*_1_*δ*[*White *- *Black*]*a *+ *λ*_2_*δ*[*Mexican*]*a*),

where *δ [X] = 1 *if *X *and *0 *otherwise.

The final models were estimated using the STATA 8.0 (College Station, TX) **svypoisson **command (log-linear model), which is appropriate for complex survey estimation. The sample weight, cluster, and strata variables suggested by the NHANES III analytical guidelines were used to adjust the estimates for the sample design. The variance was estimated by the linearization method [29]. The *R*_0 _and *A *and their confidence intervals were estimated using the **nlcom **command for non-linear transformations of the regression parameters.

**Table 3:** Comparison of force of infection for different viruses for selected* age ranges.

Virus	Force of infection (per 100 persons per year)	Ages modeled	Study sample	Citation
	
Measles	20	11–17	Lit. review – misc. sources	[18]
Mumps	12	11–17	Lit. review – misc. sources	[18]
Rubella	10	11–17	Lit. review – misc. sources	[18]
Varicella	6	≥ 10	Convenience sample	[30]
CMV†	3.1 and 3.5	16–40	Hospital-based	[10]
CMV	1.8	12–49	Population-based	Current study
HSV-2	0.84	≥ 12	Population-based	[31]
Hepatitis B	0.15	6–39	Population-based	[32]

*Ages were selected to be roughly comparable with the ages we modeled; in general, young children were not selected for comparison because they often had much higher forces of infection. †Patients were recruited from 2 different hospitals.

## Pre-publication history

The pre-publication history for this paper can be accessed here:


